# PEGylated exenatide injection (PB-119) improves beta-cell function and insulin resistance in treatment-naïve type 2 diabetes mellitus patients

**DOI:** 10.3389/fphar.2023.1088670

**Published:** 2023-09-14

**Authors:** Xu Liu, Ling Song, Yuanhui Zhang, Haiyan Li, Cheng Cui, Dongyang Liu

**Affiliations:** ^1^ Drug Clinical Trial Center, Peking University Third Hospital, Beijing, China; ^2^ Beijing Key Laboratory of Cardiovascular Receptors Research, Beijing, China; ^3^ Center of Clinical Medical Research, Institute of Medical Innovation and Research, Peking University Third Hospital, Beijing, China

**Keywords:** PB-119, beta-cell function, insulin resistance, oral minimal model, type 2 diabetes

## Abstract

**Objective:** PB-119, a PEGylated exenatide injection, is a once-weekly glucagon-like peptide-1 receptor agonist. In the present study, we aimed to evaluate the effects of PB-119 on insulin resistance and beta-cell function in Chinese patients with type 2 diabetes mellitus (T2DM) to uncover its antidiabetic characteristics.

**Methods:** A total of 36 Chinese T2DM patients were randomized to receive 25 μg and 50 μg PB-119 once weekly and exenatide (5–10 μg injected under the skin 2 times a day adjusted by the doctor) for 12 weeks. Oral mixed meal tolerance tests were conducted before the study and on Day 79. The data were fitted to estimate beta-cell function and insulin sensitivity parameters using the SAAM II package integrating the oral minimal model (OMM), which was compared with Homeostatic Model Assessment (HOMA) analysis results.

**Results:** Exenatide or PB-119 treatment, compared with their baseline, was associated with higher beta-cell function parameters (φ_b_, φ_s_ and φ_tot_), disposition index, insulin secretion rates, and a lower glucose area under the curve. High-dose PB-119 also has a higher insulin resistance parameter (SI) than the baseline, but HOMA-IR did not. For the homeostatic model assessment parameters, HOMA-IR showed no statistically significant changes within or between treatments. Only high-dose PB-119 improved HOMA-β after 12 weeks of treatment.

**Conclusion:** After 12 weeks of treatment, PB-119 decreased glycemic levels by improving beta-cell function and insulin resistance.

## 1 Introduction

Type 2 diabetes mellitus (T2DM) is a complex metabolic disease characterized by concomitant insulin resistance and impaired beta-cell function that results in chronic hyperglycemia (Hameed et al., 2015). Insulin resistance is the inability of a cell, tissue, or organism to respond appropriately to a given dose of insulin. β-cell function is described as the capacity of pancreatic β-cells to produce, store and release insulin in sufficient amounts to maintain euglycemia (Hannon et al., 2018). Progressive deficiencies in insulin resistance or impaired beta-cell compensation for insulin resistance characterize the progression from normal glucose tolerance (NGT) to impaired glucose tolerance (IGT), and type 2 diabetes is characterized (Chang et al., 2006). Despite the availability of many antidiabetic drugs, it is difficult to slow or stop disease progression and achieve appropriate glycemic targets. Insulin resistance and impaired beta-cell function are the two major pathophysiologic abnormalities of T2DM (DeFronzo et al., 1979). Therefore, quantifying insulin resistance and beta-cell function is crucial for revealing pathological feature changes in patients and estimate the effectiveness of anti-diabetic agents.

The oral minimal model (OMM) is a powerful method to quantify insulin resistance and beta-cell function. It is based on the minimal model and has been validated with two gold standard methods, the clamp and the minimal model (Cobelli et al., 2014). Comparing with gold standard methods, OMM is more convenient and reliably mimics the normal physiological state. In recent years, an increasing number of studies have assessed the effects of novel drugs on insulin resistance and beta-cell function using OMM. Visentin, R (Visentin et al., 2020) and Schiavon, M (Schiavon et al., 2021) utilized OMM to investigate the therapeutic efficacy of SAR425899, a dual glucagon-like peptide-1 receptor/glucagon receptor agonist. They demonstrated that SAR425899 improved glucose levels by significantly enhancing both beta-cell function and insulin resistance. Using OMM, we also successfully demonstrated the hypoglycemic effect of a novel dipeptidyl-peptidase-IV inhibitor by enhancing beta-cell function in Chinese T2DM patients (Liu et al., 2021).

Glucagon-like peptide-1 receptor agonists (GLP-1 RAs) stimulate hyperglycemia-induced insulin secretion, decrease glucagon secretion, inhibit the hunger center, and delay gastric emptying, preventing large post-meal glycemic increments and reducing calorie intake and body weight (Nauck et al., 2021). According to American Diabetes Association 2022 recommendations, GLP-1 RAs with or without metformin is an acceptable starting treatment for individuals with T2DM who have with or are at high risk for atherosclerotic cardiovascular disease, heart failure, and/or chronic kidney disease (American Diabetes, 2022). Exenatide, the first-in-class GLP-1 RAs, exhibits many favorable features, such as being very effective at lowering blood sugar levels, promoting weight loss, and reducing the risk of cardiovascular disease. For example, exenatide showed dose-dependent progressive weight reduction in sulfonylurea-treated individuals with T2DM over a duration of 30 weeks, with a loss of 1.6 ± 0.3 kg from baseline in the 10 μg exenatide treatment at the end of the clinical trial (Buse et al., 2004). Similar findings were obtained by Blonde L et al., exenatide decreased body weight by 2.1 ± 0.2 kg from baseline at week 30, and by 4.4 ± 0.3 kg at week 82. In addition, a statistically significant improvement in cardiovascular risk factors like triglycerides, total cholesterol, and high-density lipoprotein cholesterol were also seen at week 82 (Blonde et al., 2006). However, the original subcutaneous formulation of exenatide has a short terminal half-life of 2.4 h, requiring twice daily injections, which limits its routine use (Parkes et al., 2013; Ryan et al., 2013). PEGylated exenatide injection (PB-119) is a covalent attachment of polyethylene glycol (PEG) to exenatide that prolongs the half-life of exenatide in the circulation by increasing the relative molecular mass and decreasing the renal clearance rate. In a phase II randomized, double-blind, parallel, placebo-controlled study, the efficacy and safety of PB-119 were evaluated. The results showed that PB-119 had superior efficacy compared with placebo, safety, and was well tolerated over 12 weeks in treatment-naïve T2DM patients (Ji et al., 2021).

In this study, the aim was to provide further insights into the quantification of PB-119 effects on insulin resistance and beta-cell function and to compare it with the active comparator exenatide in Chinese T2DM patients.

## 2 Methods

### 2.1 Data source

Data from a randomized, open, and positive-controlled study were used to assess the safety, tolerability, pharmacokinetics, and pharmacodynamics of the repeated subcutaneous administration of PB-119 in naïve T2DM (NCT:03059719). Chinese subjects with naïve T2DM were recruited if they met the criteria as follows: diagnosed with T2DM during the last 5 years, age between 18 and 75 years; a body mass index of between 19 and 35 kg/m^2^, body weight for men ≥50 kg, body weight for women ≥45 kg; HbA1c between 7.0% and 10%, fasting plasma glucose level between 7.0 and 13.0 mmol/L. Subjects were excluded from the study if they met one of the following criteria: I type diabetes patients, acute complications of diabetes that occurred within 6 months prior to screening; allergic to exenatide, the study drug or any of its excipients (citric acid, mannitol, m-cresol); patients with significant cardiovascular, hepatic, renal, gastrointestinal, immune, or neurologic system diseases; serum creatine >the upper limit of normal; TBIL >1.5 times of the upper limit of normal; ALT or AST >2 times of the upper limit of normal; diastolic blood pressure >95 mm Hg, systolic blood pressure>160 mm Hg; triglyceride ≥5 mmol/L; patients who received any anti-diabetic agents; patients who received diet pills within the last 3 months or any medications that may have affected the outcomes of the clinical trial; patients with a history of alcohol abuse or drug abuse; patients showing electrocardiographic abnormality; hemorrhage or donation of more than 400 mL blood within 8 weeks; or tumor.

In a multiple-dose study, 36 subjects with naïve T2DM were recruited and allocated to cohorts based on their order of entry into the study. There were three groups, including 25 μg and 50 μg PB119 once weekly and exenatide (5–10 μg injected under the skin 2 times a day adjusted by a doctor) for 12 weeks. A total of 29 subjects finished the trial. Each subject underwent two mixed meal tolerance tests (MMTT) on Day −1 (baseline) and Day 79. Blood samples were collected at 0, 15, 30, 60, 120, 180, and 240 min for the detection of plasma glucose and serum insulin and C-peptide levels. Table 1 shows that the baseline demographic characteristics of these patients were similar among the groups. Seven participants withdrew from the clinical trial. Three patients withdrew from the Exenatide group due to excessive fasting blood glucose, having a myocardial infarction, or failing to meet the inclusion criteria. Three patients withdrew from the PB119 25 μg group due to excessive fasting blood glucose and hyperlipemia requirements. One patient voluntarily withdrew from the PB119 50 μg group.

**TABLE 1:** Baseline characteristics of volunteers.

	Exenatide	PB119 25 μg	PB119 50 μg
Number	9	9	11
Sex (Male/Famale)	3/6	4/5	5/6
Age (year)	52.11 ± 4.14	53.22 ± 7.07	53.55 ± 6.42
Height (cm)	162.51 ± 9.25	164.12 ± 9.24	161.72 ± 9.36
Weight (kg)	70.42 ± 8.95	75.82 ± 12.96	71.38 ± 8.34
BMI	26.7 ± 3.09	27.98 ± 2.59	27.33 ± 2.71
FPG (mmol/L)	9.79 ± 1.77	8.53 ± 1.06	9.41 ± 1.91
FSI (µU/mL)	8.97 ± 3.58	14.51 ± 8.18	10.09 ± 4.11
FSC (ng/mL)	2.21 ± 0.61	2.71 ± 0.95	2.69 ± 0.67
HbA1c%	8.56 ± 0.76	8.43 ± 0.6	8.14 ± 0.84

Values are reported as mean (standard deviation).

FPG: fasting plasma glucose; FSI: fasting serum insulin; FSC: fasting serum C-peptide.

### 2.2 Sensitivity index and beta-cell function assessment

SAAM II 2.3.1.1 software was acquired from the Epsilon Group. The assessment method was performed according to a previous study (Liu et al., 2021). Briefly, two separate OMM models, the glucose minimal model and the C-peptide minimal model, were used to assess SI and beta-cell function before and after treatment with PB-119 or exenatide. The oral glucose minimal model describes the interaction of glucose and insulin in the body and provides an estimate of the SI parameter, an index quantifying the ability of insulin to suppress endogenous glucose production and promote glucose disposal. The C-peptide minimal model describes the plasma C-peptide concentration in relation to the observed changes in glucose concentration and provides an estimate of the total insulin secretion rate (ISR), φ_b_, φ_s_, and φ_tot_. φ_b_ was the basal beta-cell function and was calculated from fasting plasma C-peptide and glucose data as the ratio of basal secretion per unit of basal glucose concentration. φ_s_ was the static responsivity parameter. φ static quantifies the delayed (by a time constant T) provision of new releasable insulin above a certain threshold level. φ_tot_ was the total beta-cell function. The disposition index (DI_tot_) was defined as φ_tot_ × SI. The homeostatic model (HOMA) was used to assess insulin resistance by HOMA-IR and beta-cell function by HOMA-β. HOMA-IR and HOMA-β were calculated by R 3.6.2 and R Studio 1.1 software. HOMA-IR was defined as fasting glucose concentration (mmol/L) × fasting insulin concentration (μU/mL)/22.5. HOMA-β was defined as 20 × fasting insulin concentration (μU/mL)/(fasting glucose concentration (mmol/L)-3.5).

### 2.3 Statistical analysis

For the primary outcomes, the model parameters were calculated using SAAM II 2.3.1.1 software. The patient characteristics and the response to the mixed meal tolerance test were reported as median (25th-75th) percentiles. Two-way analysis of variance (two-way ANOVA) was utilized to determine the differences between treatments and visits for normally distributed and homogenous variables. The *post hoc* analysis was conducted using the estimated marginal means (EMMs) test adjusted by Bonferroni. Otherwise, a Kruskal–Wallis test was used for the nonparametric test, and Bonferroni’s test was used to adjust Dunnett’s *t*-test for multiple comparisons.

## 3 Results

### 3.1 Comparable effects of PB-119 and exenatide on plasma levels of glucose, insulin, and C-peptide

**FIGURE 1:**
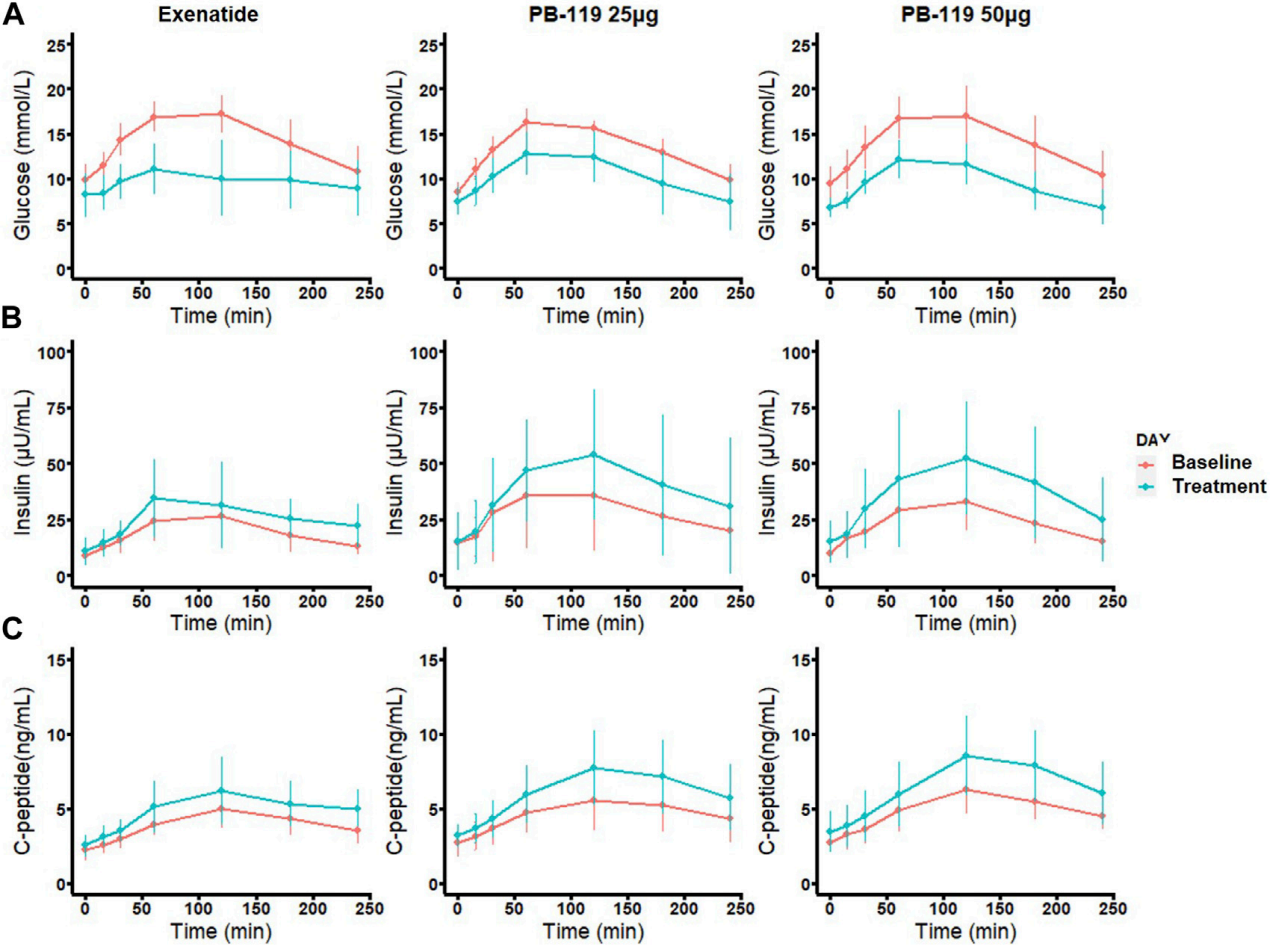
Time curve of plasma levels of glucose, insulin, and C-peptide in patients at baseline and treated with exenatide and PB-119 at 25 and 50 μg. In a multiple-dose study, 29 subjects with naïve T2DM were received subcutaneous injection 25 μg, 50 μg PB119 once weekly or exenatide (5–10 μg injected, 2 times a day adjusted by a doctor) for 12 weeks. Each subject underwent two mixed meal tolerance tests (MMTT) on Day −1 (baseline) and Day 79. Blood samples were collected at 0, 15, 30, 60, 120, 180, and 240 min for the detection of plasma glucose and serum insulin and C-peptide levels **(A-C)**. The red lines represented glucose, insulin, and C-peptide levels before exenatide, PB-119 25 μg, and PB-119 50 μg treatment. The green lines represented the glucose, insulin, and C-peptide levels following exenatide, PB-119 25 μg, and PB-119 50 μg treatment. Data were shown as mean ± SD.

To assess the efficacy of PB-119 in T2DM patients, we made a head-to-head comparison of PB-119 and exenatide on plasma levels of glucose, insulin, and C-peptide at baseline and after 12 weeks of treatment. Figure 1 displays the postprandial plasma glucose, insulin, and C-peptide concentrations over time. Table 2 shows the area under the curve (AUC) of plasma glucose, insulin, and C-peptide, as well as the basal (at t = 0 min) concentrations for all groups.

**TABLE 2:** Glucose, insulin and C-peptide outcomes.

Outcomes	Visit	Exenatide	PB119 25 μg	PB119 50 μg
Gb (mmol/L)	Baseline	10.24 [8.42,11.30]	8.44 [7.90,9.42]	9.58 [8.50,10.88]
	Treatment	8.58 [6.18,10.49]*	7.68 [6.80,8.48]	6.45 [6.08,7.50]**
Ib (μU/mL)	Baseline	9.49 [7.06,10.15]	13.56 [9.72,16.76]	10.61 [6.95,11.40]
	Treatment	8.03 [6.91,14.27]	10.29 [9.14,13.02]	12.44 [8.52,17.95]
Cpb (ng/mL)	Baseline	2.17 [1.76,2.66]	2.57 [2.07,2.99]	2.68 [2.38,3.21]
	Treatment	2.29 [1.94,3.07]	3.04 [2.87,3.42]	3.29 [2.34,4.46]
GAUC (mmol/L*min)	Baseline	3477.90 [3156.00, 3986.85]	3391.88 [3103.43, 3454.12]	3502.12 [3194.70, 3797.93]
	Treatment	2522.25 [1820.40, 2768.17]**	2585.55 [2070.97, 2964.30]**	2213.55 [2046.00, 2658.04]**
IAUC (μU/mL*min)	Baseline	4562.85 [3545.25, 5135.32]	5612.18 [3728.18, 8218.95]	5965.12 [4719.90, 7035.79]
	Treatment	6764.10 [4610.70, 8152.58]	8418.00 [5745.53,11079.00]	8870.85 [5323.42,13372.39]
CAUC (ng/mL*min)	Baseline	952.88 [ 778.73, 963.98]	1119.00 [ 880.72, 1236.98]	1162.05 [1077.82, 1288.09]
	Treatment	1304.17 [ 911.32, 1388.70]	1271.70 [1244.10, 1759.12]*	1440.83 [1311.34, 1941.71]**

Values are reported as medians [interquartile range].

Gb: Fasting plasma glucose change from baseline; GAUC: area under glucose concentration—time curve; Ib: Fasting serum insulin change from baseline; IAUC: area under insulin concentration—time curve; Cpb: Fasting serum C-peptide change from baseline; CAUC: area under C-peptide concentration—time curve *p < 0.05, **p < 0.01, compared with their baselines.

For glucose, administration of exenatide or PB-119 treatment resulted in a decreased mean glucose concentration at each time point on the curve than their baseline. The lower basal glucose concentrations were shown on Day 79 than baseline after exenatide (8.58 [6.18,10.49] vs. 10.24 [8.42,11.30] mmol/L, *p* < 0.05) or PB-119 50 μg (6.45 [6.08,7.50] vs. 9.58 [8.50,10.88] mmol/L, *p* < 0.01) treatment. The glucose AUC (GAUC) were lower on Day 79 than they were at baseline after treatment with exenatide (2522.25 [1820.40, 2768.17]. vs. 3477.90 [3156.00, 3986.85] mmol/L × min, *p* < 0.01), PB-119 25 μg (2585.55 [2070.97, 2964.30] vs. 3391.88 [3103.43, 3454.12] mmol/L × min, *p* < 0.01), or PB-119 50 μg (2213.55 [2046.00, 2658.04] vs. 3502.12 [3194.70, 3797.93] mmol/L × min, *p* < 0.01). For insulin and C-peptide, administration of exenatide or PB-119 treatment resulted in increased mean concentrations at each time point on the curve than their baseline. However, there were no statistically significant changes in the concentration of basal insulin (Ib), the concentration of basal C-peptide (Cpb), or the insulin AUC(IAUC). When comparing C-peptide AUC (CAUC) at Day 79 and baseline, PB-119 25 μg (1271.70 [1244.10, 1759.12] vs. 1119.00 [880.72, 1236.98] ng/mL × min, *p* < 0.05), or PB-119 50 μg (1440.83 [1311.34, 1941.71] vs. 1162.05 [1077.82, 1288.09] ng/mL × min, *p* < 0.01) had a greater CAUC at Day 79, but exenatide exhibited no significant changes.

### 3.2 Similar effects of PB-119 and exenatide on insulin action and beta-cell responsivity

**FIGURE 2:**
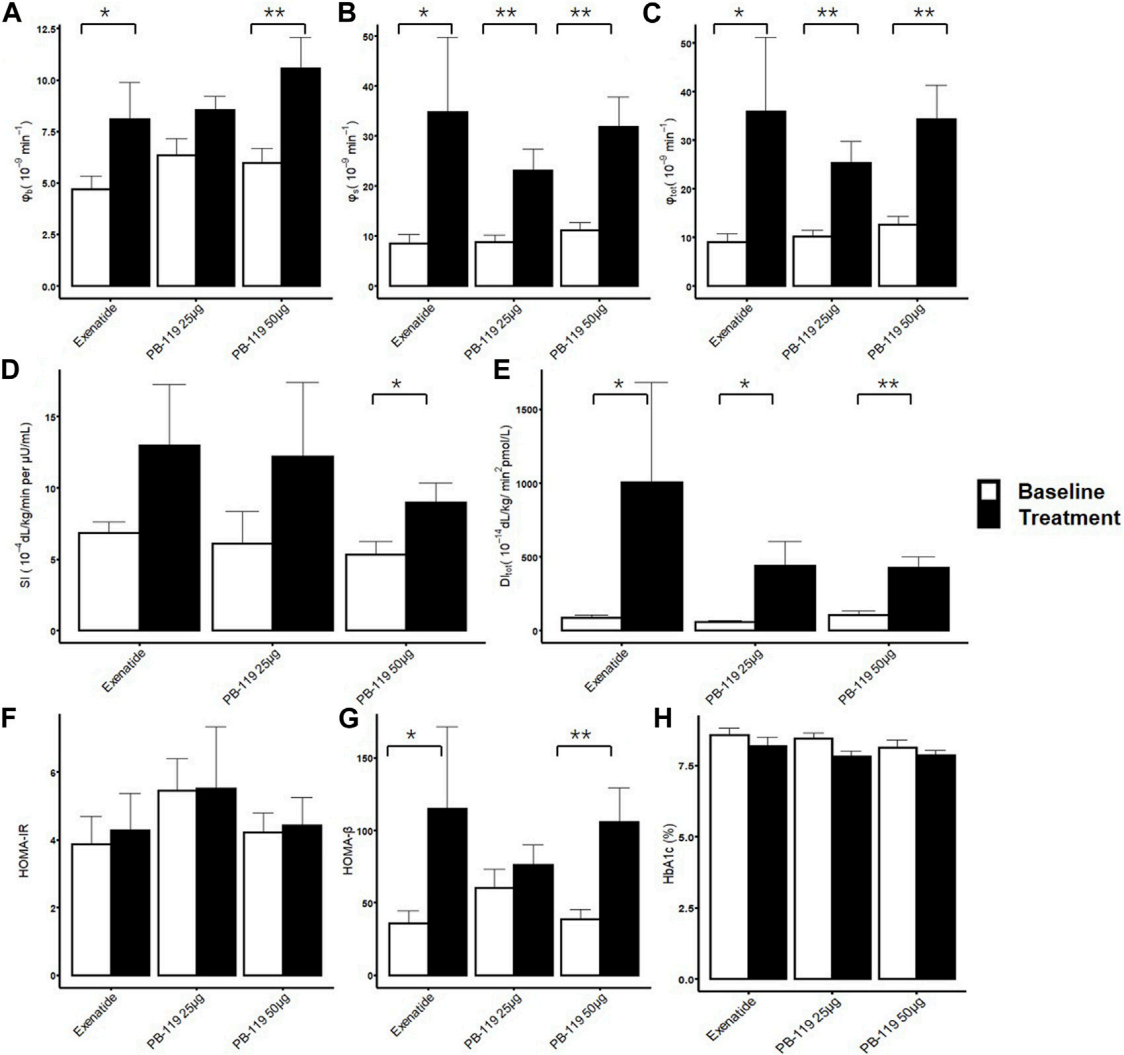
Insulin resistance and beta-cell function assessment by oral minimal model. The treatment effect of exenatide and PB-119 on insulin resistance and beta-cell function were determined by oral minimal model and homeostatic model assessment (HOMA). The results showed that both exenatide and PB-119 could improve beta-function parameters, such as φ_b,_ φ_s,_ φ_tot_,HOMA-β and DI **(A–C, E, G)**. But only PB-119 showed that significantly increased SI while exenatide did not **(D)**. For HOMA-IR and HbA1c, no difference was found in all groups **(F, H)**. SI: insulin sensitivity; φ_b_: basal beta-cell function; φ_s_: static beta-cell function; φ_tot:_ total beta-cell function; DI: total disposition index; HOMA-IR: insulin resistance by HOMA; HOMA-β: beta-cell function by HOMA. Open bars and solid bars were the baseline and treatment with exenatide or PB-119. Data were shown as mean ± SE.*p < 0.05, **p < 0.01, baseline compared with treatment.

We then assessed the effect of PB-119 and exenatide treatment on insulin resistance and beta-cell function by using OMM and HOMA. Figure 2 shows that high-dose PB-119 significantly increased SI on Day 79 than baseline (9.00 ± 1.35 vs. 5.31 ± 0.93 × 10^−4^ dL/kg/min per μU/mL, *p* < 0.05). Increased SI also could be observed in low-dose PB-119 (12.21 ± 5.22 vs. 6.10 ± 2.26 × 10^−4^ dL/kg/min per μU/mL, *p* > 0.05) and exenatide (12.95 ± 4.33 vs. 6.85 ± 0.75 × 10^−4^ dL/kg/min per μU/mL, *p* > 0.05) although these differences were not significant. No significant differences in HOMA-IR were observed between visits or groups.

Comparing Day 79 visits and baseline, the basal beta-cell function indexes, φ_b_ and HOMA-β showed improvements in high-dose PB-119 (φ_b_:10.55 ± 1.51 vs. 5.96 ± 0.73 × 10^−9^ min^−1^, *p* < 0.05. HOMA-β:105.83 ± 23.37 vs. 38.63 ± 6.94, *p* < 0.05) and exenatide (φ_b_: 8.08 ± 1.79 vs. 4.71 ± 0.62 × 10^−9^ min^−1^, *p* < 0.05. HOMA-β: 115.03 ± 56.47 vs. 35.70 ± 8.59, *p* < 0.05). The greater increases of φ_s_ and φ_tot_ were observed in low-dose PB-119 (φ_s_: 23.09 ± 4.22 vs.8.79 ± 1.31 × 10^−9^ min^−1^, *p* < 0.05, φ_tot_:25.27 ± 4.43 vs. 10.09 ± 1.40 × 10^−9^ min^−1^, *p* < 0.05.), high-dose PB-119 (φ_s_: 31.82 ± 6.03 vs.11.17 ± 1.47 × 10^−9^ min^−1^, *p* < 0.05, φ_tot_:34.35 ± 6.95 vs. 12.55 ± 1.76 × 10^−9^ min^−1^, *p* < 0.05.) and exenatide (φ_s_: 34.73 ± 14.97 vs. 8.43 ± 1.88 × 10^−9^ min^−1^, *p* < 0.05, φ_tot_:35.86 ± 15.25 vs. 9.02 ± 1.72 × 10^−9^ min^−1^, *p* < 0.05). No significant differences in φ_s_ and φ_tot_ were found between exenatide and PB-119.

The DI significantly increased on Day 79 than baseline in low-dose PB-119 (440.03 ± 165.09 vs. 58.87 ± 10.28 dL/kg/min^2^ pmol/L, *p* < 0.05), high-dose PB-119 (423.75 ± 76.52 vs. 105.11 ± 26.89 dL/kg/min^2^ pmol/L, *p* < 0.05) and exenatide (1006.94 ± 677.63 vs. 87.40 ± 16.04 dL/kg/min^2^ pmol/L, *p* < 0.05), but no significant difference was found between exenatide or PB-119.

No changes were found in HbA1c between visits or groups over 12 weeks.

### 3.3 Similar effects of PB-119 and exenatide on insulin secretion rate

**FIGURE 3:**
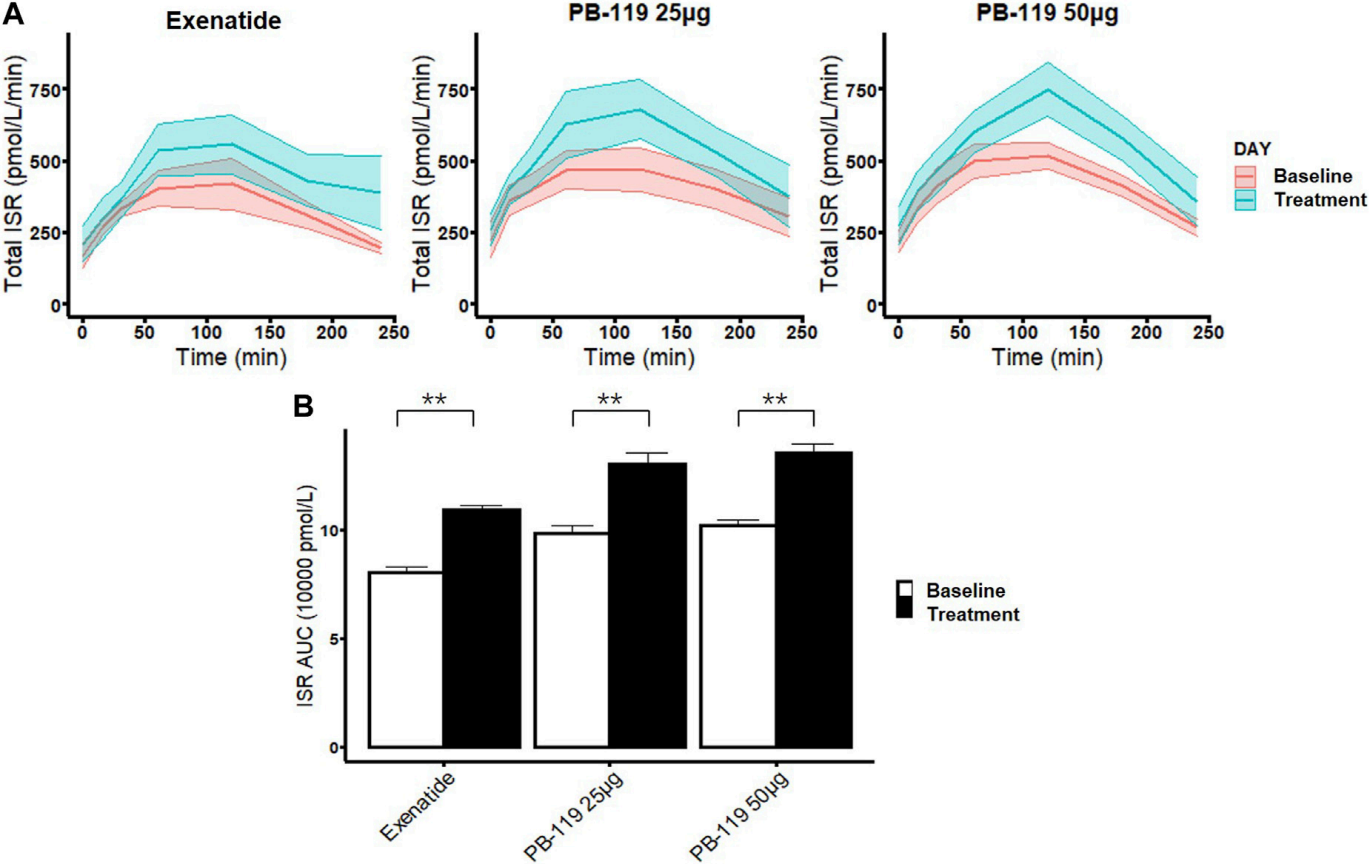
The time curve and the area under the curve of insulin secretion rate in patients at baseline and treated with exenatide and PB-119 at 25 and 50 μg Both the PB-119 and exenatide groups had significantly higher ISR AUCs than their baseline values. The red lines represented glucose, insulin, and C-peptide levels before exenatide, PB-119 25 μg, and PB-119 50 μg treatment. The green lines represented the glucose, insulin, and C-peptide levels following exenatide, PB-119 25 μg, and PB-119 50 μg treatment **(A)**. Data were shown as means ± SE. ISR AUC: the area under the curve (AUC) of insulin secretion rate **(B)**. Data were shown as median and 95% confidential interval. *p < 0.05, **p < 0.01, baseline compared with treatment.

To further assess chronic effect of PB-119 on beta-cell function, we compared effects of treatments of PB-119 and exenatide on insulin secretion rate (total ISR) and AUC of ISR (Figure 3). Administration of exenatide or PB-119 treatment resulted in an increased ISR curve than their baseline. Comparing with their baseline values, significantly higher ISR AUCs were observed in the low-dose PB-119 (13.05 ± 0.47 vs. 9.81 ± 0.37, *p* < 0.05), high-dose PB-119 (13.55 ± 0.38 vs. 10.20 ± 0.23, *p* < 0.05) and exenatide (10.91 ± 0.21 vs. 8.03 ± 0.27, *p* < 0.05).

## 4 Discussion

PB-119 is a covalent attachment of PEG to exenatide, prolonging the half-life of exenatide in circulation by increasing the relative molecular mass and reducing the renal clearance rate (Ji et al., 2021), making it more convenient for patients to use. In this study, data from a 12-week, phase Ib study in naïve T2DM patients were used to quantify the effects of PB-119, a long-acting GLP-1 RA, on glycemic control and to compare it with exenatide. The results showed that PB-119, like exenatide, had a good anti-diabetic effect in Chinese T2DM patients, exhibiting decreased GAUC and Gb and increased mean insulin and C-peptide concentration in the MTT study (Figure 1; Table 2).

Several clinical and mechanistic studies have indicated that GLP-1 RAs could improve both insulin resistance and beta-cell function. For example, Sarkar G et al. discovered that 6 months of exenatide treatment decreased insulin resistance in type 1 diabetic patients (Sarkar et al., 2014). Gedulin BR et al. showed that exenatide treatment could improve the insulin sensitivity index by 224% higher than the control group, and increased beta-cell mass×insulin sensitivity index in insulin-resistant obese rats during 6 weeks (Gedulin et al., 2005). In this study, we used OMM and HOMA to assess PB-119 effects on insulin resistance and beta-cell function and to compare it with exenatide. The OMM results showed that both exenatide and PB-119 could significantly increase the beta-cell function parameters (φ_b_, φ_s_, and φ_tot_) and the ISR over 12 weeks, while only high-dose PB-119 had a higher SI after treatment. The HOMA results showed that both exenatide and PB-119 significantly increased HOMA-β but not HOMA-IR. PB-119 and exenatide may improve SI and beta-cell function via numerous pathways, such as slow gastric emptying, suppressing glucagon secretion, anti-inflammation, weight loss, and lipid metabolism improvement eta (Wang et al., 2021). In an oral glucose tolerance test, Gastaldelli A (Gastaldelli et al., 2016) demonstrated that acute exenatide administration improves hepatic insulin resistance, hepatic glucose uptake and insulin’s antilipolytic effect, decreases endogenous glucose production, adipose insulin resistance and plasma free fatty acid levels. GLP-1 RA improved lipid metabolism diseases by regulating certain miRNAs involved in lipid metabolism and stimulating lipid metabolic enzyme activation (Yaribeygi et al., 2019). GLP-1 also has anti-inflammatory properties and has been shown to reduce macrophage activity in adipose tissue as well as the expression and synthesis of IL-6, TNF-a, MCP-1, and NF-kB (Bednarz et al., 2022; Mehdi et al., 2023). The underlying mechanism is still not fully understood and further research is warranted.

In this study, 50 μg of PB-119 caused a substantial increase in SI, but exenatide did not. However, it should be pointed that the mean value was increased by two times compared with its baseline. The reason might be the difference of short- and long-acting GLP-1 RAs. Gastaldelli, A (Gastaldelli et al., 2014).reported the similar results with us. Taspoglutide, as a long-acting GLP-1 RA, but not exenatide, decreased hepatic insulin resistance during 24 weeks. For pharmacokinetics, short-acting GLP-1 RAs had intermittent exposure that changes between C_max_ and C_ss,trough_ with negligible drug concentrations in between. Comparing with short-acting GLP-1 RAs, long-acting GLP-1 RAs had a continuous exposure, with a smaller fluctuation in plasma drug concentrations. For the drug effect, short-acting GLP-1 RAs reduced postprandial glucose levels, while long-acting GLP-1 RAs showed a significantly more effective for HbA1c, fasting plasma glucose levels, and body weight (Gastaldelli et al., 2014; Guo, 2016; Huthmacher et al., 2020). Therefore, long-acting GLP-1 RAs may be better in the improvement of insulin resistance than short-acting GLP-1 RAs.

Insulin resistance and insulin secretion are linked because insulin resistance is compensated for by increased insulin secretion, revealing a hyperbolic function. DI is insulin sensitivity multiplied by beta-cell function. It is a constant, which means that when an individual’s beta-cells respond to an increase in insulin resistance by increasing insulin secretion appropriately, DI is unchanged, and normal glucose tolerance is retained. In contrast, the individual develops glucose intolerance if there is an insufficient compensatory increase in beta-cell function in response to increased insulin resistance (Cobelli et al., 2007). DI of both PB-119 and exenatide had more than 4 times higher than their baseline, predicting more powerful glucoregulatory control.

ISR provides important information about how a person’s endocrine system can use insulin to control glucose levels by describing the absolute insulin output, which depends on how well β cells respond (Abohtyra et al., 2022). Direct observations of ISR are not possible, as insulin has a very short half-life and undergoes a first-pass extraction in the liver; therefore, C-peptide concentration is the most common quantification method for ISR (Magkos et al., 2022). C-peptide and insulin are released simultaneously and in the same quantity. C-peptide has a longer half-life than insulin and undergoes less degradation in the liver (Defronzo, 2009). The oral C-peptide minimal model can estimate ISR and beta-cell function parameters and has been used in many studies. In this study, Figure 3 showed that both exenatide and PB-119 significantly increased the ISR curve and the AUC of ISR. The results were in line with expectations because GLP-1 RAs could stimulate the beta cells to secrete insulin.

The correlation coefficient indicated that φ_b_ has a strong correlation with HOMA-β, but not with other beta-cell function parameters. High-dose PB-119 improved SI after treatment, but HOMA-IR did not. The discrepancy might cause the difference between HOMA and OMM, as HOMA only considered basal plasma glucose, insulin, or C-peptide levels. However, OMM considered their levels in the stimulus state. OMM provided us with more information on beta-cell function than only in the basal state.

There were no changes in HbA1c between visits or groups over 12 weeks, but exenatide and PB-119 led to similar decreases of 0.39% and 0.62%, respectively. The hypoglycemic effect of exenatide has been proven in several clinical trials; therefore, a statistically nonsignificant result could merely be due to inadequate sample size. A PB-119 phase II study showed that the mean differences in HbA1c in the treatment groups were −0.72%, −1.18%, and −1.02% in the 75 μg, 150 μg, and 200 μg PB-119 groups after placebo adjustment, respectively, at the end of 12 weeks (Ji et al., 2021). Based on Gb, GAUC, and HbA1c findings, PB-119 had a notable antidiabetic effect even below the phase II clinical dose.

Our study also had some limitations. The current analysis was carried out based on a phase-I study with a modest sample size, and larger studies are required to confirm the findings. OMM was estimated in the shoulder of minimal model. Similar to minimal model, it has some defects (Krudys et al., 2006; Ibrahim et al., 2019). Three individuals in the exenatide group could not be estimated by OMM. We tried to optimize the model, but the model could not be minimized. The flat glucose curve and few sampling points may be the reason for the failure.

Collectively, we showed that PB-119, a long-acting GLP-1 RAs, could exert an antidiabetic effect by improving beta-cell function, ISR, and insulin resistance.

## Data Availability

The original contributions presented in the study are included in the article/Supplementary Materials, further inquiries can be directed to the corresponding authors.
